# Supplementary material for: A nomogram for predicting bladder dysfunction in patients with type 2 diabetes mellitus: a retrospective study
Source: PeerJ. 2025 Jan 22;13:e18872. doi: 10.7717/peerj.18872 (PMC11760200; doi:10.7717/peerj.18872)
Supplement: Supplemental Information 3 [file peerj-13-18872-s003.doc]

Assigned values of categorical variables.

| **Variables** | **Description/Recoding** |
| --- | --- |
| Gender | Male=1,Women=2 |
| Occupation | Employed =1, Unemployed=2, Other = 3 |
| Marital status | Unmarried=1, Married=2, Divorced=3, Widowed= 4 |
| Education level | Illiterate=1, Primary school=2, Middle school=3, Senior high school=4  University and above=5 |
| Insurance type | Self-Pay=1, Insured=2 |
| Oral hypoglycemic agents | No=0, Yes=1 |
| Insulin injection | No=0, Yes=1 |
| Mecobalamin | No=0, Yes=1 |
| DPN | No=0, Yes=1 |
| Diabetic retinopathy | No=0, Yes=1 |
| Diabetic nephropathy | No=0, Yes=1 |
| Hypertension | No=0, Yes=1 |
| Coronary heart disease | No=0, Yes=1 |
| Urinary frequency | No=0, Yes=1 |
| Urinary urgency | No=0, Yes=1 |
| Polyuria | No=0, Yes=1 |
| Nocturia | No=0, Yes=1 |
| Dysuria | No=0, Yes=1 |
| UI | No=0, Yes=1 |
| UR | No=0, Yes=1 |
| UA/CR | Normal=1, Microalbuminuria=2, Macroalbuminuria=3 |
| Nit | No=0, Yes=1 |
| Pro | No=0, Yes=1 |
| Glu | No=0, Yes=1 |
| Ket | No=0, Yes=1 |
| UBG | No=0, Yes=1 |
| OB | No=0, Yes=1 |
| DBD | DBD=0, Yes=1 |

***Note:*** DPN, Diabetic Peripheral Neuropathy; UI, Urinary Incontinence; UR, Urinary Retention;UA/CR , Urine Microalbumin/Creatinine Ratio; Nit , Nitrite ; Pro , Protein ; Glu , Glucose ; Ket , Ketones ; UBG , Urobilinogen ; OB , Occult Blood.
