# Supplementary material for: A nomogram for predicting bladder dysfunction in patients with type 2 diabetes mellitus: a retrospective study
Source: PeerJ. 2025 Jan 22;13:e18872. doi: 10.7717/peerj.18872 (PMC11760200; doi:10.7717/peerj.18872)
Supplement: Supplemental Information 4 [file peerj-13-18872-s004.docx]

**STROBE Statement**—Checklist of items that should be included in reports of

***case-control studies***

|  | **Item No** | **Recommendation** | | Reported on Page # |
| --- | --- | --- | --- | --- |
| **Title and abstract** | 1 | (*a*) Indicate the study’s design with a commonly used term in the title or the abstract | | #1 Title |
|  |  | (*b*) Provide in the abstract an informative and balanced summary of what was done and what was found | | #1-2 Abstract |
| **Introduction** | | | |  |
| Background/rationale | 2 | Explain the scientific background and rationale for the investigation being reported | | #3 Introduction Paragraph 1-2 |
| Objectives | 3 | State specific objectives, including any prespecified hypotheses | | #3-4 Introduction Paragraph 3 |
| **Methods** | | | |  |
| Study design | 4 | Present key elements of study design early in the paper | | #4 Methods  2.1Study population |
| Setting | 5 | Describe the setting, locations, and relevant dates, including periods of recruitment, exposure, follow-up, and data collection | | #4 Methods  2.1Study population & #8  3.1Study Participants |
| Participants | 6 | (*a*) Give the eligibility criteria, and the sources and methods of case ascertainment and control selection. Give the rationale for the choice of cases and controls | | #4 Methods  2.1Study population |
|  |  | (*b*) For matched studies, give matching criteria and the number of controls per case | | Not applicable |
| Variables | 7 | Clearly define all outcomes, exposures, predictors, potential confounders, and effect modifiers. Give diagnostic criteria, if applicable | | #5-7 Methods  2.3 Study Variables |
| Data sources/ measurement | 8* | For each variable of interest, give sources of data and details of methods of assessment (measurement). Describe comparability of assessment methods if there is more than one group | | #5-7 Methods  2.3 Study Variables |
| Bias | 9 | Describe any efforts to address potential sources of bias | | #7-8 Methods  2.4 Data Analysis |
| Study size | 10 | Explain how the study size was arrived at | | #5 Methods  2.2 Sampling |
| Quantitative variables | 11 | Explain how quantitative variables were handled in the analyses. If applicable, describe which groupings were chosen and why | | #6 Methods  2.6 Data Analysis |
| Statistical methods | 12 | (*a*) Describe all statistical methods, including those used to control for confounding | | #7-8 Methods  2.6 Data Analysis |
|  |  | (*b*) Describe any methods used to examine subgroups and interactions | | Not applicable |
|  |  | (*c*) Explain how missing data were addressed | | Missing values were not included in the study |
|  |  | (*d*) If applicable, explain how matching of cases and controls was addressed | | Not applicable |
|  |  | (*e*) Describe any sensitivity analyses | | Not applicable |
| **Results** | | | |  |
| Participants | 13* | (a) Report numbers of individuals at each stage of study—eg numbers potentially eligible, examined for eligibility, confirmed eligible, included in the study, completing follow-up, and analysed | | #8 Results  3.1 Study Participants |
|  |  | (b) Give reasons for non-participation at each stage | | Figure 1 |
|  |  | (c) Consider use of a flow diagram | | Figure 1 |
| Descriptive data | 14* | (a) Give characteristics of study participants (eg demographic, clinical, social) and information on exposures and potential confounders | | Table 1, Table 2, Table 3 |
|  |  | (b) Indicate number of participants with missing data for each variable of interest | | Not applicable |
| Outcome data | 15* | Report numbers in each exposure category, or summary measures of exposure | | #8 Results  3.2 Comparison of Relevant Factors in Patients with T2DM  &Table 1, Table 2, Table 3 |
| Main results | 16 | (*a*) Give unadjusted estimates and, if applicable, confounder-adjusted estimates and their precision (eg, 95% confidence interval). Make clear which confounders were adjusted for and why they were included | | #8 Results  3.3 Logistic Regression &Table 4 |
|  |  | (*b*) Report category boundaries when continuous variables were categorized | | Not applicable |
|  |  | (*c*) If relevant, consider translating estimates of relative risk into absolute risk for a meaningful time period | | Not applicable |
| Other analyses | 17 | Report other analyses done—eg analyses of subgroups and interactions, and sensitivity analyses | | Not applicable |
| **Discussion** | | |  | |
| Key results | 18 | Summarise key results with reference to study objectives | | #10 Discussion  Paragraph 1 |
| Limitations | 19 | Discuss limitations of the study, taking into account sources of potential bias or imprecision. Discuss both direction and magnitude of any potential bias | | #14-15 Discussion  Paragraph 11 |
| Interpretation | 20 | Give a cautious overall interpretation of results considering objectives, limitations, multiplicity of analyses, results from similar studies, and other relevant evidence | | #8-11 Discussion  Paragraph 2-10 |
| Generalisability | 21 | Discuss the generalisability (external validity) of the study results | | Not applicable |
| **Other information** | | |  | |
| Funding | 22 | Give the source of funding and the role of the funders for the present study and, if applicable, for the original study on which the present article is based | | #Title Page |

*Give information separately for cases and controls.

**Note:** An Explanation and Elaboration article discusses each checklist item and gives methodological background and published examples of transparent reporting. The STROBE checklist is best used in conjunction with this article (freely available on the Websites of PLoS Medicine at <http://www.plosmedicine.org/>, Annals of Internal Medicine at

<http://www.annals.org/>, and Epidemiology at http://www.epidem.com/). Information on the STROBE Initiative is available at <http://www.strobe-statement.org>.
