# Supplementary material for: A nomogram for predicting bladder dysfunction in patients with type 2 diabetes mellitus: a retrospective study
Source: PeerJ. 2025 Jan 22;13:e18872. doi: 10.7717/peerj.18872 (PMC11760200; doi:10.7717/peerj.18872)
Supplement: Supplemental Information 5 [file peerj-13-18872-s005.doc]

### Appendix 1. Instructions for using the nomogram: A tool for predicting diabetic bladder dysfunction (DBD) in type 2 diabetes patients.

1. **Variables included in the nomogram**

This nomogram includes 11 variables designed to predict a specific clinical risk or outcome. These variables are: HbA1c (Glycated Hemoglobin), PCP-2h (Postprandial 2-hour C-Peptide), DPN (Diabetic Peripheral Neuropathy), TCO2 (Total Carbon Dioxide), PAB (Prealbumin), T-Bil (Total Bilirubin), I-Bil (Indirect Bilirubin), IgE (Immunoglobulin E), URBC (Urinary Red Blood Cells), UI (Urinary Incontinence), and UR (Urinary Retention). Each variable corresponds to a specific scale in the nomogram, allowing the assignment of a score based on its observed value. The cumulative scores from these variables facilitate the estimation of the clinical risk or outcome, offering a practical tool for decision-making in a clinical context.

1. **Steps for using the nomogram**

(1) Determine the score for each variable

The value for each variable can be found on its corresponding scale, which indicates the score. For example:

- HbA1c: The HbA1c value ranges from 0 to 18, with different scores corresponding to specific values. For instance, if the HbA1c value is 10, the corresponding score is approximately 9.
- DPN: If the patient has DPN (choose "Yes"), the corresponding score is approximately 23. If not (choose "No"), the score is 0.
- PCP-2h: The PCP-2h value ranges from 0 to 90, with specific values corresponding to different scores. For example, if the PCP-2h value is 10, the corresponding score is approximately 10.
- TCO_2_ : For a TCO_2_ value of 20, the corresponding score is approximately 7.5.
- For other variables, such as PAB, T-Bil, and I-Bil, scores can be found on the corresponding scales based on the specific values.

(2) Calculate the total score

The total score, referred to as "Total Points," is obtained by summing the scores of each variable. For example, if the scores for all variables total 160, locate the corresponding position for 160 on the "Total Points," scale.

(3) Convert to risk probability

Locate the corresponding "risk" scale below the "total points" scale. This scale is used to convert the total score into the corresponding risk probability. For example, when the total score is 160, the corresponding risk probability on the "risk" scale is approximately 55%.

(4) Interpretation of each variable

- HbA1c: A measure of average blood glucose levels over the past 2-3 months. High values indicate prolonged hyperglycemia, which may increase the risk of diabetic complications.
- PCP-2h: The postprandial 2-hour C-peptide level reflects insulin secretion. High values may suggest excessive insulin secretion or impaired insulin action, and are associated with diabetes or other metabolic disorders.
- DPN: DPN caused by diabetes-induced damage to the nervous system, is characterized by symptoms such as numbness and pain in the hands and feet.
- TCO_2_, PAB, T-Bil, I-Bil: These variables reflect different aspects of the patient's health: acid-base balance (TCO_2_), nutritional/liver function (PAB), liver function and biliary system status (T-Bil, I-Bil). Changes in these variables affect the score and, consequently, the risk assessment.
- IgE: Associated with allergic reactions and immune function. High levels may suggest an allergic response or immune system dysfunction.
- URBC: The number of red blood cells in the urine. Elevated values may indicate kidney dysfunction or abnormal urine quality.
- UI: Urinary incontinence refers to the involuntary leakage of urine, typically linked to bladder dysfunction or neurological disorders.
- UR: Urinary retention, where the bladder is unable to fully empty, is also associated with bladder dysfunction or neurological issues.

These variables are represented on the nomogram with corresponding scales, where each scale corresponds to a score. The scores are used to assess the patient's overall health and the risk of diabetic bladder dysfunction (DBD).

(5) Clinical application example

In clinical practice, a comprehensive risk assessment was conducted for a 65-year-old diabetic patient, Mr. Zhang. Mr. Zhang has a 10-year history of diabetes and recently developed symptoms of foot numbness. After a professional diagnosis, he was confirmed to have DPN, which was recorded as "Yes" in the assessment. To obtain a more comprehensive understanding of Mr. Zhang's health status, multiple physiological indicators were collected, including HbA1c of 10%, PCP-2h of 20 ng/mL, TCO_2_ of 20 mmol/L, PAB of 200 mg/L, T-Bil of 15μmol/L, I-Bil of 10 μmol/L, IgE of 200 IU/mL, URBC of 10 cells/μL, and he denied symptoms of UI and UR.

Based on the nomogram calculation, the patient's scores for each variable are as follows: DPN 23, HbA1c 9, PCP-2h 19, TCO_2_ 7, PAB 27, T-Bil 45, I-Bil 23, IgE 4, URBC 0.6, UI 0, and UR 0, giving a total score of 167.6. On the "Total Points" scale, a score of 167.6 corresponds to a risk probability of approximately 68%. This high risk probability indicates that the patient may face a significantly elevated risk of DBD.

Therefore, the physician should first perform further examinations (such as urodynamic studies, bladder ultrasound, and neurological evaluation) to confirm the diagnosis. Based on the results, a personalized treatment plan should be developed, and early interventions should be implemented. Additionally, multidisciplinary collaboration should be considered to optimize bladder health management, effectively addressing diabetic bladder dysfunction and its related complications.

**3. Conclusion**

This nomogram provides physicians with a multifactorial risk assessment tool, enabling rapid estimation of a patient's risk probability through simple calculations, thereby supporting personalized medical decision-making. The integration of multiple factors in this model aids in the accurate assessment and prediction of a patient's health status in clinical practice.
